# Do Intestinal Unicellular Parasites Have a Role in the Inflammatory and Redox Status among the Severely Obese?

**DOI:** 10.3390/antiox11112090

**Published:** 2022-10-23

**Authors:** Jana Caudet, María Trelis, Susana Cifre, Gabriela Tapia, José M. Soriano, Regina Rodrigo, Juan F. Merino-Torres

**Affiliations:** 1 Department of Endocrinology and Nutrition, University and Polytechnic Hospital La Fe, 46026 Valencia, Spain; 2 Joint Research Unit on Endocrinology, Nutrition and Clinical Dietetics, Health Research Institute Hospital La Fe-University of Valencia, 46026 Valencia, Spain; 3 Parasite & Health Research Group, Area of Parasitology, Department of Pharmacy and Pharmaceutical Technology and Parasitology, University of Valencia, 46010 Valencia, Spain; 4 Food & Health Lab, Institute of Materials Science, University of Valencia, 46980 Valencia, Spain; 5 Pathophysiology and Therapies for Vision Disorders, Principe Felipe Research Center (CIPF), 46012 Valencia, Spain; 6 Joint Research Unit on Rare Diseases, CIPF-Health Research Institute Hospital La Fe, 46012 Valencia, Spain; 7 Centre for Biomedical Research on Rare Diseases (CIBERER), Instituto de Salud Carlos III, 28029 Madrid, Spain; 8 Department of Physiology, University of Valencia, 46010 Valencia, Spain; 9 Department of Medicine, Faculty of Medicine, University of Valencia, 46010 Valencia, Spain

**Keywords:** obesity, lipoinflammation, oxidative stress, antioxidant defense, *Blastocystis*, *Giardia intestinalis*, *Dientamoeba fragilis*, food antioxidants

## Abstract

The diagnosis of obesity comprises subjects with totally different phenotypes and metabolic profiles. Systemic inflammation and oxidative stress derived from the white adipose tissue are suggested as the link between this disease and the development of insulin resistance and metabolic comorbidities. The presence of unicellular eukaryotic parasites colonizing the human gut ecosystem is a common circumstance, and yet their influence on the inflammatory and redox status of the obese host has not been assessed. Herein, a set of inflammatory and redox biomarkers were assessed together with a parasitological analysis of 97 severely obese subjects. Information was also collected on insulin resistance and on the antioxidant composition of the diet. The global prevalence of intestinal unicellular parasites was 49.5%, with *Blastocystis* sp. the most prevalent protozoan found (42.3%). Colonized subjects displayed a higher total antioxidant capacity and a trend towards higher extracellular superoxide dismutase activity, regardless of their insulin resistance status, along with lower reduced glutathione/oxidized glutathione (GSH/GSSG) ratios in plasma in the insulin-resistant subgroup. No changes in malondialdehyde levels, or in inflammatory cytokines in plasma, were found in regard to the colonization status. In conclusion, enteric eukaryotic unicellular parasites may play an important role in modulating the antioxidant defenses of an obese host, thus could have beneficial effects with respect to the development of systemic metabolic disorders.

## 1. Introduction

Obesity is increasing worldwide, and it is becoming a major public health burden. The lack of impactful behavioral or pharmacological interventions will only aggravate the situation soon [1]. Numerous research papers have established a relationship between a situation of low-level systemic inflammation (which is a recognized hallmark of obesity), the development of the metabolic syndrome, and the related chronic pathologies [2,3,4]. Lipoinflammation is a phenomenon that takes place among insulin-resistant obese individuals, in which immune cells inhabiting the white adipose tissue increase the delivery of pro-inflammatory cytokines (TNFα, IL-6) to the bloodstream [5]. The most remarkable feature of these cells is the phenotypic switch of the macrophage towards a pro-inflammatory (M1) profile, which unbalances the physiological M1/M2 ratio [6,7,8]. This enhances the insulin resistance phenomenon and promotes metabolically unhealthy obesity [9,10,11]. Some studies suggest that gut dysbiosis can start the macrophage phenotypic polarization by augmenting the release of lipopolysaccharides that are derived from Gram-negative bacteria of the dysbiotic intestinal ecosystem [12,13], coupled to the increased intestinal barrier permeability that is associated with obesity [14,15,16]. Conversely, the role of oxidative stress in the development of many disorders is also the focus of recent interest. Oxidative stress in eukaryotic cells results from the imbalance between the production of reactive oxygen species and the effects of antioxidant molecules, but the exact mechanisms determining the loss of antioxidant capacity in the progression of disease remain elusive. Several studies show that obesity is also associated with heightened oxidative stress [17,18,19,20], as well as comorbidities such as insulin resistance [21,22,23,24], metabolic syndrome [25] and non-alcoholic fatty liver disease (NAFLD) [26,27], closely interrelating this circumstance with lipoinflammation [28,29]. On the other hand, some researchers have focused on the immunomodulatory properties of eukaryotic parasites on the human host. Helminths can trigger a natural immune response of Th2-phenotype, and prevent the excessive inflammatory response of the human host: the Th2 response promotes a reduction in the macrophagic activation, and a polarization towards the anti-inflammatory M2 phenotype, which protects from lipoinflammation and from insulin resistance [30]. Accordingly, a direct relationship between the eradication of these organisms in developed countries and the occurrence of chronic metabolic disorders, such as metabolic syndrome and diabetes mellitus, has been described [31,32]. Although less well-known, some parasitic protozoa are also proven to modify the immune response of their hosts through alterations in the production of specific cytokines [33]. Some data show that even intestinal parasites that cannot penetrate the mucosa can cause intestinal inflammation. The parasite *Blastocystis* sp. can both impair intestinal barrier integrity [34,35] and induce the expression of pro-inflammatory cytokines by colonocytes and macrophages (proven in vitro [36] and in a mice model [37]). Therefore, it can modulate the inflammatory pathways of its host, yet we still lack information regarding its systemic inflammatory effects. However, in subjects affected by irritable bowel syndrome, this parasite can activate the mucosal immune system and enhance systemic low-grade inflammation [38]. *Giardia intestinalis*, another intestinal parasite, can also disrupt the duodenal epithelial barrier by altering its tight junctions [39], but this infection is devoid of signs of overt intestinal inflammation [40]. Conversely, systemic disorders caused by unicellular parasites are situations of enhanced oxidative stress [41,42], with oxygen reactive species generated by the host through the activation of the macrophage respiratory burst [43]. However, research on the influence of intestinal parasites on systemic oxidative stress is still scarce [44]. 

Based on the evidence, it is reasonable to suspect that intestinal unicellular eukaryotes can alter both the systemic oxidative and inflammatory status of their hosts. We previously reported [45] an unexpectedly high prevalence of intestinal unicellular eukaryotes (*Blastocystis* sp., *Dientamoeba fragilis* and *Giardia intestinalis*) within the gut ecosystem of severely obese subjects. In this study, we also stated a significant difference in the Homeostatic Model Assessment of Insulin Resistance (HOMA-IR) levels regarding the colonization status, with lower indexes in those subjects’ harboring parasites, and suggested that their presence affects the host’s metabolism. In a subgroup of this severely obese population, we also detected variations in the richness and diversity of the intestinal bacterial communities that were associated with the specific parasitic species: the presence of *Blastocystis* and/or *D. fragilis* was related to higher diversity indexes and to a potential beneficial effect on metabolic comorbidities [46]. The purpose of the present paper is to further explore the correlation between these intestinal parasites, and the underlying inflammation and oxidative stress, at a systemic level.

We determined plasmatic levels of three of the best-known pro-inflammatory cytokines (TNF-α, IL-1β and IL-6), whose high concentrations are commonly associated with an underlying inflammatory disorder, including obesity [47] and insulin resistance [48]. We also analyzed the levels of fibrinogen and C-Reactive Protein (C-RP) and studied the following redox biomarkers: (i) malondialdehyde (MDA), a plasmatic indicator of oxidative damage; (ii) glutathione (GSH), glutathione disulfide (GSSG) and plasmatic GSH/GSSG ratio (considered a good indicator of the redox state in living beings [49]); (iii) nitrites and nitrates in plasma (NOX)—their levels in biological fluids rise during inflammation, due to the overexpression of the inducible NO-synthase enzyme [50]; (iv) extracellular superoxide dismutase (EC-SOD or SOD-3)—its expression inversely correlates with obesity state [51]; (v) total antioxidant capacity in plasma (TAC), which is considered the sum of all the effects of individual antioxidants that are present in plasma (vitamin A, vitamin C, carotenoids, glutathione, uric acid, etc.). These biomarkers were analyzed for any correlation with colonization by intestinal parasites. As secondary aims, they were related with the presence of insulin resistance, other metabolic comorbidities, and the dietary intake of antioxidants. Additionally, we assessed plasmatic levels of immunoglobulins A (IgA) and E (IgE), as IgA has been used as a diagnostic tool in giardiasis [52,53], and levels of IgE may become modified by some intestinal parasites [54], and have been described as augmented in human giardiasis [55,56,57]. Finally, we performed a white blood cell count to obtain the absolute eosinophil levels, since eosinophilia has been described in some intestinal parasitic diseases, such as dientamoebiasis [58] and giardiasis [59], and is related to chronic urticaria, which is a symptom of blastocystiasis [60].

## 2. Materials and Methods

### 2.1. Design of the Study

This investigation is part of a larger single-center and cross-sectional study conducted from August 2018 to February 2020 in obese subjects attending the Endocrinology and Nutrition Service of University and Polytechnic Hospital La Fe (Valencia, Spain). Informed consent was obtained from all participants, after being informed about the aim of the study, risks, and implications of their participation in it, as well as the treatment and confidentiality of the data. This study was approved by the Ethics Committee for Drug Research of the University and Polytechnic Hospital La Fe (23 May 2018), ensuring that the fundamental principles established by the Helsinki Declaration, data protection and bioethics were respected.

### 2.2. Study Population

Data from 97 subjects (58 women and 39 men) with a mean age of 47.9 years were gathered. Inclusion criteria were: (a) age from 18 to 65 years old; (b) BMI > 40 kg/m^2^ or >35 kg/m^2^ in coexistence with significant comorbidity; (c) no sustained weight loss with non-surgical measures. Exclusion criteria were: (a) diagnosis of oncologic disease or active oncologic process in the last 5 years; (b) acute gastrointestinal symptoms; (c) autoimmune or chronic gut inflammatory diseases; (d) systemic chronic inflammatory disease; and (e) therapy with any systemic glucocorticoid or immunosuppressive drug during the 30 days before the study visit. Every participant was put on a standard hypocaloric diet, defined as a caloric input between 20 and 25 kcal/kg total weight/day [61], and received dietary advice to bring their diet as close as possible to the Mediterranean diet model [62].

### 2.3. Clinical Interview and Anthropometric Assessment

Every patient held interviews with an endocrinologist and a nutritionist, in which relevant information was recorded regarding medical history and epidemiological data. To assess the quality of the diet followed, each participant filled a five-day dietary register. Using the DIAL program (Department of Nutrition UCM, Alce Ingeniería S.L., Madrid, Spain) [63], we calculated the daily intakes of the antioxidant nutrients: vitamin A, vitamin C, vitamin E, omega 3 and omega 6 fatty acids, selenium, copper, zinc and manganese. International cut-offs were applied as a reference to consider whether the participants fulfilled or not the daily nutritional needs [64]. Furthermore, the patients were analyzed for the following metabolic comorbidities: hypertension, type 2 diabetes mellitus or pre-diabetes, dyslipidemia, and hyperuricemia. Metabolic syndrome was diagnosed according to the National Cholesterol Education Program (NCEP) Adult Treatment Panel III (ATP III) definition [65]. The presence of hepatic steatosis suggestive of NAFLD was assessed by means of ultrasonography. Finally, anthropometric measurements were obtained in light clothes and with no shoes after an overnight fast. The same calibrated wall height rod was employed for every participant. The multifrequency bioelectrical impedance scale Inbody 770^®^ (Biospace Co. Ltd., Seoul, Korea) [66,67] was used to register weight, body mass index (BMI, calculated as kg/m^2^) and the following bioimpedance estimations: total body water (L), percentage of fat mass (% FM), percentage of fat-free mass (% FFM), percentage of skeletal muscle mass (% SKM), phase angle, and visceral fat area (VFA, cm^2^).

### 2.4. Stool Sampling and Molecular Parasitological Analysis

Each participant provided three stool samples collected on alternate days in REAL MiniSystems with Total-fix fixative (Durviz, Valencia, Spain) for conservation and concentration by centrifugation. Stool DNA was extracted from 200 µL of the concentrate with the QIAamp DNA Stool Mini Kit (QIAGEN, Hilden, Germany) according to the manufacturer’s instructions. Extracted DNA was stored at −20 °C until its use in parasitological analysis.

Multiplex real-time PCR (qPCR) for the detection and identification of intestinal parasites was performed using a commercially available kit, Allplex GI-Parasite Assay (GIPPA) (Seegene, Seoul, South Korea) [68,69]. This panel consists of a simultaneous real qPCR for the detection of six common protist parasites in humans, such as *Giardia duodenalis*, *Entamoeba histolytica*, *Cryptosporidium* spp., *Blastocystis hominis*, *Dientamoeba fragilis* and *Cyclospora cayetanensis*. Amplification was performed on the CFX96 Real-Time PCR System (Bio-Rad, Marnes-la-Coquette, France), and managed with the CFX Manager IVD 1.6 software, in a 25 µL reaction volume containing 20 µL reaction mix (5 µL Primers Mom, 5 µL Anyplex PCR MM (EM2), 8 µL of DNase/RNase-free water and 2 µL of internal control DNA (provided)) and 5 µL of DNA. Negative (DNase/RNase-free water) and positive controls were included in each assay. The results were analyzed using the Seegene Viewer V3 software optimized for multiplex assays. Samples were considered positive for specific parasites if the cycle threshold (Ct) was ≤43 cycles according to the manufacturer’s instructions.

### 2.5. Laboratory Tests

Blood samples were obtained under clinically stable conditions, and with the participant being asymptomatic for inflammatory or infectious complaints, on the same day as fecal samples. Blood was collected after an overnight fast using heparin as the anticoagulant and processed within 1 h after collection. It was centrifuged for ten minutes at 800× *g* to obtain the top yellow plasma layer, which was stored at −80 °C for further analysis.

The following biochemical and hematological parameters were analyzed by the Clinical Analysis Service of the Hospital La Fe: (i) fasting glucose and insulin, to calculate the HOMA-IR index; an insulin cut-off of above of 3.8 was established as indicative of insulin resistance [70]; (ii) plasmatic levels of leptin; (iii) plasmatic levels of C-RP, ferritin and ceruloplasmin; (iv) plasmatic levels of vitamin A, vitamin E and uric acid (part of the antioxidant scavengers of the redox system); (v) circulating total levels of immunoglobulins A and E; and (vi) white blood cell count, with the absolute neutrophil and eosinophil levels expressed as count × 10^3^/µL.

An Enzyme-Linked Immunosorbent Assay (ELISA) for human pro-inflammatory cytokines (IL-1β, IL-6 and TNF-α) in plasma was performed using commercial sandwich kits (Invitrogen^®^, Thermo Fisher Scientific, USA), as follows: human IL-1β uncoated ELISA^®^, human IL-6 uncoated ELISA^®^, and human TNF- α uncoated ELISA^®^. Sandwich ELISA was carried out on a CorningTM CostarTM 9018 96-well plate, following the manufacturer’s instructions. The absorbance was measured by optical density at 450 nm in the iMark™ Microplate Absorbance Reader (Bio-Rad^®^, Hercules, CA, USA). Plasma IL-1β, IL-6 and TNF-α levels were expressed as pg/mL.

Finally, the oxidative stress biomarkers were analyzed in plasma samples by the Analytical Unit of the Health Research Institute Hospital La Fe. Malondialdehyde (MDA) as a marker of lipid peroxidation, and glutathione in reduced (GSH) and oxidized (GSSG) states, were measured by the Ultra-Performance Liquid Chromatography (UPLC)–multiple reaction monitoring–Mass Spectrometry (MS) method [71] using a Waters Acquity UPLC system equipped with an Acquity UPLC HSS T3 column (1.8 µm, 2.1 mm × 100 mm; Waters). The MS analysis was performed using a Waters Xevo TQ-XS mass spectrometer with an electrospray ionization source working in the positive-ionization mode. Levels were expressed as plasma concentration in ng/mL.

Stable metabolites of nitric oxide (NOX), nitrites and nitrates were measured in plasma by the spectrophotometric Griess reaction using nitrate reductase, as previously described [72], and levels were expressed in µM. The amount of plasmatic SOD-3 independent of the type (Cu/Zn, Mn, Fe) and the TAC of plasma were measured with a commercial kit (Superoxide Dismutase Assay Kit and Antioxidant Assay Kit, Cayman Chemicals, Ann Arbor, MI, USA) following the manufacturer’s protocols. The enzymatic colorimetric assays were carried out in a 96-well plate and the absorbance was measured by optical density in the iMark™ Microplate Absorbance Reader (Bio-Rad^®^, Hercules, CA, USA) at 450 nm for SOD and 750 nm for TAC. SOD activity was expressed as U/mL and TAC was expressed as mM.

### 2.6. Statistical Analysis

The data obtained are shown as absolute frequency (%) for qualitative variables. For quantitative variables, the values are given as mean ± standard deviation for normally distributed variables and as median (1st and 3rd quartiles) for non-normally distributed ones. The normality of the data was checked using the Kolmogorov–Smirnov test. To compare proportions between groups, the Chi^2^ test was performed. Mean group values were compared using the T test and ANOVA test for parametric variables and the Wilcoxon rank sum test with continuity correction for non-parametric variables. Lineal regression models were used to correlate the results from redox biomarkers with continuous variables of interest. A *p*-value < 0.05 was considered to indicate statistical significance. All analyses were performed using the statistical software R (4.0 version, Vienna, Austria, 2013) [73].

## 3. Results

### 3.1. Population Description

The mean BMI was 45.6 ± 6 kg/m^2^ with eighty-three (86.5%) of the participants displaying type III obesity in terms of the WHO classification [74]. Active smoking was present in 21 subjects (21.6%). The most frequent metabolic comorbidities were dyslipidemia (75.3%), hepatic steatosis (75%), hypertension (55.7%), hyperuricemia (49.5%) and type 2 diabetes mellitus (32%). Metabolic syndrome (ATP-III definition) was present in 55.7% of cases. Forty-eight subjects (49.5%) were colonized with an intestinal unicellular eukaryote by multiplex qPCR. The most frequent species was *Blastocystis* sp. (42.3%), followed by *D. fragilis* (10.3%) and *G. intestinalis* (9.3%), with six cases combining *Blastocystis* sp./*D. fragilis*, five cases of *Blastocystis* sp./*G. intestinalis*, and one case colonized by the three species. No cases were detected of *Entamoeba histolytica*, *Cryptosporidium* spp. or *Cyclospora cayetanensis*, with species included in the same multiplex panel.

Epidemiological and clinical information is shown in Table 1, clustered by the presence of an intestinal unicellular eukaryote. These two groups were comparable in terms of age, type of obesity, smoking condition, and prevalence of metabolic comorbidities, except for a lower prevalence of type 2 diabetes among colonized subjects. Additionally, participants harboring an intestinal parasite showed lower HOMA-IR indexes (5.2 ± 2.3 vs. 7.1 ± 4.7, *p* = 0.021) (Table 1), along with a trend towards lower metabolic syndrome and lower hepatic steatosis. These results agree with the ones presented in our previous work [45].

After clustering by colonization status, the anthropometric results obtained by electrical bioimpedance also revealed no differences between groups (results shown in Supplementary Table S1).

### 3.2. Inflammatory Biomarkers

We gathered information regarding the following inflammatory parameters: total leucocytes, neutrophils and eosinophils recount, plasmatic levels of C-RP, ferritin and ceruloplasmin, and cytokine levels of TNF-α, IL-1β and IL-6. The participants presented slightly elevated levels of C-RP (8.4 ± 7.0 mg/L) and ferritin (143.4 ± 124.2 ng/mL); TNF-α plasmatic levels were highly variable, with a median of 25.4 (9.1, 105.7) pg/mL. IL-1β and IL-6 levels were exceptionally low or undetectable; thirty-seven samples yielded IL-1β values under the detection limit, while regarding IL-6, we obtained positive results in only three samples.

**Figure 1:**
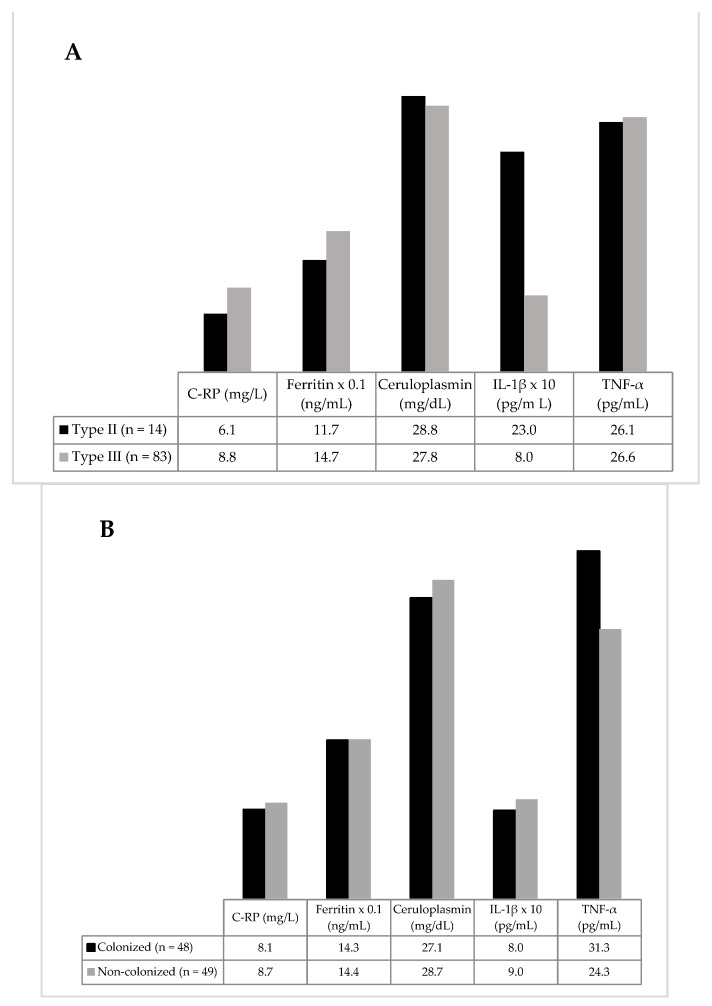
Inflammatory biomarkers clustered by type of obesity (**A**) and by colonization status (**B**). Mean levels of C-reactive protein (C-RP), ferritin and ceruloplasmin are shown, along with median levels of Il-1β and TNF-α. No statistical differences were found between groups.

Slightly higher C-RP and ferritin levels were found in type III than in type II obese subjects, but without reaching statistical significance, due to the small number of participants in the last group (Figure 1A).

**Figure 2:**
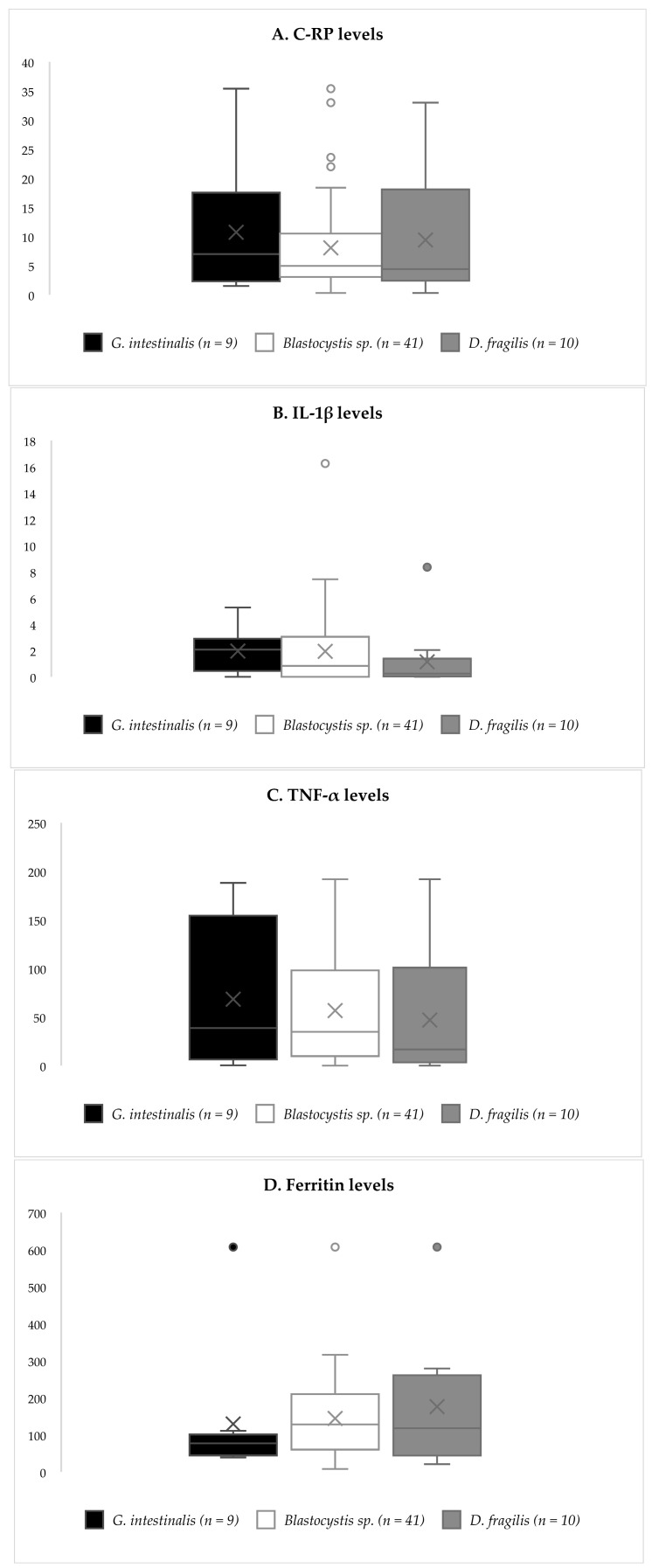
Inflammatory biomarkers comparing colonized subjects in regard to parasitic species. (**A**) C-RP (mg/L) levels boxplots. (**B**) IL-1β (pg/mL) levels boxplots. (**C**) TNF-α (pg/mL) levels boxplots. (**D**) ferritin (ng/mL) levels boxplots.

When analyzing the inflammatory biomarkers as regards colonization status, again, no differences between groups were detected (Figure 1B). However, when participants were classified according to their specific colonization, a non-significant trend towards lower levels of C-RP was observed among *Blastocystis* sp.-positives (Figure 2).

Regarding comorbidities, significantly higher levels of C-RP were rendered by insulin-resistant subjects, and significantly higher levels of ferritin among those suffering from NAFLD; no other differences were detected in cytokine levels as regards metabolic comorbidities (Table 2).

Regarding the white blood cell count, the absolute count of eosinophils was significantly higher in colonized vs. non-colonized subjects, as well as the absolute count of monocytes, the absolute counts of total leucocytes, neutrophils, and lymphocytes being equivalent (Table 3). 

When sub-analyzing by specific parasitic species, these differences only remained significant when comparing those non-colonized with the group of *Blastocystis* sp.-positives (Table 3). Finally, a trend towards lower IgE levels in non-colonized participants existed, without correlation with specific parasitic species (Table 3). We observed that values above the normal range were shown by 11 and 29 participants, for IgA and IgE, respectively.

### 3.3. Redox Biomarkers

We collected information concerning plasmatic levels of GSH, GSSG, TAC, MDA, SOD-3 and NOX. No significant differences were found between groups when clustering by sex, age, smoking condition or type of obesity, even though a trend existed towards higher levels of NOX and lower levels of SOD-3 in type III compared with type II obese subjects. We classified participants according to their plasmatic levels of vitamins A and E (low, normal or high) and compared the concentrations of redox biomarkers between them. Only significant differences were found in TAC in regard to plasmatic vitamin E, with higher results of this parameter with increasing plasmatic amounts of vitamin E (Table 4). 

Table 5 shows redox biomarkers in regard to colonization status. Remarkably, subjects colonized by an intestinal parasite showed a significantly higher TAC (*p* = 0.009), a lower GSH/GSSG index (*p* = 0.034) and a non-significant trend towards higher levels of SOD-3. Plasmatic levels of the antioxidants vitamin A and E were similar, regardless of the colonization status.

**Figure 3:**
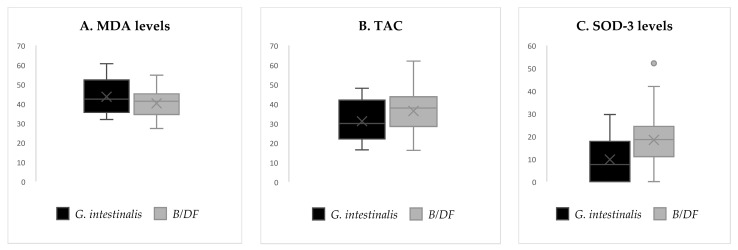
Some redox biomarkers among colonized subjects in regard to parasitic species. (**A**) MDA (ng/mL) levels boxplots. (**B**) TAC (mM) boxplots. (**C**) SOD-3 (U/mL) levels boxplots. *B/DF* = *Blastocystis* sp. and/or *D. fragilis* positives after excluding subjects co-infected with *G. intestinalis*.

After sub-analyzing by specific parasitic species, when comparing *G. intestinalis*-positives with *Blastocystis* sp. and/or *D. fragilis*-positives, we detected a reduced antioxidant response among the formers, shown by trends towards lower SOD-3 activity and lower TAC (Table 5 and Figure 3). This finding suggests a stronger antioxidant response among subjects colonized by *Blastocystis* sp. and/or *D. fragilis* than by *G. intestinalis*, and therefore, a differential pattern between parasitic species.

According to their metabolic comorbidities, insulin-resistant participants showed significantly higher GSH/GSSG ratios, SOD-3 activity and NOX levels than insulin-sensitive participants. NAFLD-positive participants presented higher GSH/GSSH ratios and NOX levels than NAFLD-negative ones (Table 6). No such results were obtained regarding type 2 diabetes or other comorbidities.

Since oxidative stress may be modified both by the presence of the intestinal parasites and the metabolic status, we grouped participants based on their colonization and the presence of insulin resistance (Table 7). Higher TAC and lower GSH/GSSG ratios persisted among colonized subjects who were insulin-resistant. Likewise, a higher TAC was found in colonized subjects with insulin-sensitivity preserved. Therefore, the upregulation of TAC was observed among colonized subjects independently of their insulin resistance status. NOX level and SOD-3 activity remained similar regardless of the colonization status. 

Finally, we constructed a lineal regression model to determine whether an association existed between the redox and inflammatory biomarkers and the levels of some biochemical and anthropometric measurements (Table 8). The results obtained show a positive and strong association between MDA levels and HOMA-IR indexes, and negative associations with leptin and vitamin A plasmatic levels. Additionally, weak positive correlations were detected between NOX levels and plasmatic ferritin, and between SOD-3 activity and plasmatic C-RP levels. Lastly, TAC showed an almost significant trend towards an inverse correlation with the % of fat mass, obtained from the bioimpedance analysis.

### 3.4. Dietary Results

Using the information gathered from 84 dietary registers, we estimated the daily intake of some nutrients that have an established role in modulating the antioxidant system (vitamin A, C, E, ω3 fatty acids, ω6 fatty acids, copper, zinc, manganese and selenium). The mean daily intakes of these nutrients are displayed in supplementary Table S2; no correlation was found between their levels and the status of parasite colonization. Among the participants, 57.1% met the daily recommended intake (DRI) of vitamin C, 34.5% for vitamin A and only 3.6% for vitamin E, while 38.1% did not meet the daily needs of any of the three. When separating the participants into several categories regarding the meeting of the DRI of these vitamins, no differences were detected in redox parameters between groups (Table S3).

## 4. Discussion

The influence of parasitic protozoa on the development of obesity and its comorbidities has been little explored. Evidence suggests a hallmark role for systemic inflammation and for oxidative stress (both originating in white adipocytes) in the progression to insulin resistance. Our understanding is still scarce regarding how protozoa disrupt host immunity and drive our inflammatory and oxidative responses. A higher prevalence of obesity was described among subjects serologically positive for *Toxoplasma gondii*, but without any causal relationship proven [75]. Additionally, latent *T. gondii* infection was associated with significantly higher rates of metabolic syndrome, higher HOMA-R indexes and worse body composition parameters in an obese adolescent population [76]. These data suggest a role for protozoa in the pathogenesis of obesity and in promoting metabolic disorders. However, to our knowledge, this is the first study focused on identifying an association between systemic low-grade inflammation, systemic oxidative stress, and intestinal eukaryotic unicellular parasites in severely obese subjects. 

In agreement with what has been widely described among the obese [77,78], participants in this study showed levels of plasmatic C-RP and ferritin slightly over the normal range. When designing this research, to limit other sources of pro-inflammatory cytokines, we avoided including patients suffering from chronic inflammatory disorders. Hence, we believe these results reflect the underlying low-grade, chronic, systemic inflammation that is linked to obesity. Accordingly, subjects with higher BMIs displayed non-significantly higher levels of C-RP. Additionally, we found meaningful positive associations between insulin resistance and higher levels of C-RP and ferritin, thus supporting the pathogenic role of lipoinflammation in developing metabolic syndrome. Fibrinogen and C-RP levels have been previously found to be strongly elevated in prediabetic subjects compared to normoglycemic individuals, a finding that we could not reproduce [79]. We obtained disappointing results with respect to the levels of plasmatic cytokines, since they were very heterogeneous and difficult to interpret, particularly IL-6; consequently, no significant differences were detected regarding the grade of obesity or the presence of metabolic comorbidities.

Concerning colonization by enteric parasites, no differences in inflammatory biomarkers could be established between groups. This reveals that the presence of these microeukaryotes was not coincident with systemic inflammation, and supports the idea that they are not harmful to the human host, but rather commensal. However, we cannot rule out a different behavior with *G. intestinalis*, which showed slightly higher levels of C-RP, but which did not reach statistical significance due to the low number of subjects harboring it. No cases of hypereosinophilia were detected, yet we described slightly higher counts of eosinophils and monocytes among colonized subjects. 

Regarding redox biomarkers, we derived some interesting results when clustering by intestinal colonization. To start with, colonized subjects displayed an augmented antioxidant response, represented by higher TAC and a trend towards higher SOD-3 activity, which is one of the main components of the antioxidant system. These results remained consistent regardless of the insulin-sensitivity status. Since this circumstance did not coexist with a drop in MDA levels, we understand it as a physiological response to counteract a chronic overload of free radicals. It is believed that, in situations of chronic exposure to oxygen reactive species (including the obese state), human tissues try to neutralize this overload by enhancing the expressions and activities of antioxidant enzymes [80]. Indeed, a weak correlation was present in this study between the levels of C-RP and the expression of SOD-3. Nevertheless, these defensive mechanisms may be progressively overwhelmed over time, driving the oxidative damage. Thereby, the expressions of the enzymes SOD, glutathione peroxidase and catalase have been inversely correlated with the obese state [51]. 

The finding of an enhanced antioxidant capacity among colonized subjects reflects a role for the intestinal protozoa in modulating the process of systemic oxidative stress. We are aware that biomarkers of oxidative damage different to the ones assessed in this study may have been increased among the colonized group. However, considering the absence of an increase in MDA or NOX levels in these participants, exposure to the parasite must have fostered an exaggerated antioxidant response in its host, to counteract the chronic overload of oxygen-reactive species. On the other hand, this colonization can promote the Th2 immune response, thus modulating the oxidative damage caused by obesity itself, and may postpone the development of insulin resistance. This explanation could justify the findings of a lower HOMA-IR index in the group of colonized participants. Contradicting this theory, other researchers have reported higher levels of oxygen peroxide, MDA, and advanced oxidative protein products in animals infected with intestinal parasites, such as *Blastocystis* sp. [81]. However, it is not possible to determine a causal relationship between colonization and the maintenance of a better metabolic profile, since harboring one of these parasites can also be regarded as an indirect marker of a host with a less active immunologic system. In this scenario, chronic colonization would be established only among subjects who develop Th2-polarized immune responses, and who are not capable of a strong inflammatory and oxidative response. These individuals would also be less prone to develop the proinflammatory status linked to obesity (determined by the macrophage M1), and therefore present with a better insulin-sensitivity profile. Be that as it may, further research on these processes is guaranteed to fill the remaining gaps in knowledge, and it is necessary to consider variables such as BMI and age, which can be responsible for the opposite outcomes obtained. Likewise, considering the redox results obtained among participants harboring *G. intestinalis*, it is probable that the specific parasitic species determines the effects exerted on the antioxidant system. The evidence accumulated from our previous work [45,46] indicates that the colonization with this protozoan may have effects on the obese host that are dissimilar from those of the other two.

Diet has a strong influence on humans’ antioxidant system through the daily intake of antioxidant molecules [82]. In this study, estimates of these intakes were generally under the DRI, without differences in regard to colonization status. This indicates that the variations in redox parameters that were found among colonized subjects were not related to diet composition. This idea was confirmed after obtaining no differences in redox biomarkers when comparing them in terms of vitamin A and E plasmatic levels. On the other hand, a relationship between higher BMI and increased levels of oxidative biomarkers has been widely described [18,83,84,85]. In this small study, no significant differences were detected when comparing type II and type III obese subjects. Central adiposity (rather that total fat mass) has been correlated with an impairment of TAC [86]. In this research, we could not reproduce this association either, since in the lineal regression, TAC levels were not significantly modified by any anthropometric parameter. Additionally, we did not find meaningful associations between tobacco abuse and higher redox biomarkers, in contrast to the common assumption that smoking is a strong positive predictor of oxidative stress [83].

Finally, in line with what has been previously published [21,22,87], signs of an underlying chronic oxidative stress were revealed in subjects suffering from traits of metabolic syndrome and insulin resistance. This was evident in the levels of NOX, which are a surrogate marker of the production of reactive nitrogen species; its levels were found increased among participants with several metabolic disorders (insulin resistance, hypertension, hyperuricemia and NAFLD). The relationship between insulin resistance and oxidative damage was also patent in the strong and positive correlation of HOMA-IR index with MDA levels, shown in the lineal regression model. Furthermore, participants with insulin resistance displayed a higher GSH/GSSH ratio and higher activity of SOD-3, indicating the compensatory response started to counteract the chronic oxidative stress overload. This last finding can be validated by results previously published [88], in which obese subjects with insulin resistance showed an increased activity of glutathione-peroxidase (the enzyme that regenerates glutathione), even though they also exhibited a lower activity of SOD, in opposition to our findings. However, it is necessary to further explore the interpretation of these redox biomarkers, since a decreased GSH/GSSG ratio in prediabetics was also described by other authors [89]. Subjects suffering from NAFLD displayed higher levels of NOX, along with a higher GSH/GSSG ratio; opposite results regarding the GSH/GSSG ratio have been rendered by other groups in subjects suffering from this disorder [27]. However, differences in the redox balance are probably influenced by the grade of hepatic inflammation and the presence of non-alcoholic steatohepatitis. Therefore, further research is needed to increase our knowledge of the pathophysiology of this disease.

## 5. Conclusions

In this research, harboring intestinal unicellular eukaryotic parasites was identified as a factor that could modulate the underlying oxidative stress of obese subjects, even though there was no direct correlation with the inflammatory status. We also identified some differences in the antioxidant response triggered in the host between parasitic species. If we grant these intestinal parasites the ability of modifying the antioxidant capacity, they could also interfere with the development of insulin resistance and the extremely prevalent metabolic disorders associated with obesity. Considering the high prevalence of enteric parasites in this population, new approaches are necessary to confirm our findings, increase our understanding of this process, and identify potentially useful targets to reverse this systemic disorder. Hopefully, this might enable us to finally unravel the pathogenic differences between metabolically healthy and unhealthy obesity. 

## Figures and Tables

**Table 1:** Epidemiological and clinical variables and HOMA-IR indexes in regard to colonization.

	Colonized		Non-Colonized		Chi ^2^ Test
	*N*	%	*N*	%	
Women	27	56.3	31	63.3	*Ns*
Type II obesity	6	12.5	8	16.3	*Ns*
Smokers	12	25	9	18.4	*Ns*
Hypertension	25	52.1	29	59.2	*Ns*
Dyslipidaemia	38	79.2	35	71.4	*Ns*
Type 2 Diabetes	11	22.9 *	20	40.8 *	***p* = 0.047**
Hepatic steatosis	31^+^	33.7	38^+^	41.3	*Ns*
Hyperuricemia	23	47.9	25	51	*Ns*
Metabolic syndrome	23	47.9	31	63.3	*Ns*
Cardiovascular disease	3	6.3	5	10.2	*Ns*
	**Mean**	**SD**	**Mean**	**SD**	***t* test**
HOMA-IR	5.2 *	2.3	7.1 *	4.7	***p* = 0.021**
Age (years)	47.9	9.7	47.8	9.7	*Ns*

*N*: number of cases; SD: standard deviation; *: statistically significant (*p* < 0.05) between groups in terms of Chi ^2^ test; *Ns*: no statistical differences found between groups; +: Missing data from several subjects in this parameter; %: adjusted to the number of subjects with information available.

**Table 2:** Inflammatory biomarkers clustered by some metabolic comorbidities.

	Insulin-Sensitivity Status ^1^		WT	Fatty Liver Disease Status ^2^		WT
	Resistant (*n* = 59)	Sensitive (*n* = 28)		NAFLD + (*n* = 69)	NAFLD - (*n* = 23)	
C-RP (mg/L)	9.8 *	5.9 *	***p* = ** **0.025**	8.5	7.9	*Ns*
Ferritin (ng/mL)	215.1	101.0	*Ns*	155.8 *	87.6 *	***p* = 0.009**
Ceruloplasmin (mg/dL)	25.5	26.0	*Ns*	26.0	28.0	*Ns*
IL-1β (pg/mL)	1.0	2.6	*Ns*	1.9	2.4	*Ns*
TNF-α (pg/mL)	12.0	49.9	*Ns*	16.9	33.4	*Ns*

^1^ After excluding 10 participants with type 2 diabetes treated with insulin. ^2^ Data regarding NAFLD was missing from 5 subjects. * Statistically significant (*p* < 0.05) between groups. WT, Wilcoxon rank sum test. *Ns*, no statistical differences found.

**Table 3:** White blood cell count and immunoglobulin levels clustered by colonization status.

	Non-Colonized (*n* = 49)	Colonized (*n* = 48)	*t* Test	Blastocystis sp. (*n* = 41)	*t* Test	G. Intestinalis (*n* = 9)	D. Fragilis (*n* = 10)
Leucocytes (×10^3^/µL)	7.5	7.8	*Ns*	7.8	*Ns*	8.4	7.3
Neutrophils (×10^3^/µL)	4.1	4.6	*Ns*	4.5	*Ns*	5.0	4.2
Lymphocytes (×10^3^/µL)	3.17	2.28	*Ns*	2.6	*Ns*	2.3	2.3
Eosinophils (×10^3^/µL)	0.16 *	0.24 *	***p* = 0.018**	0.24 *	***p* = 0.021**	0.25	0.24
Monocytes (×10^3^/µL)	0.46 *	0.61 *	***p* = 0.002**	0.60 *	***p* = 0.010**	0.58	0.58
			***WT***		***WT***		
IgA (mg/dL)	289.2	265.0	*Ns*	273.4	*Ns*	218.3	249.1
IgE (kUA/L)	47.0	70.0	*Ns*	62.0	*Ns*	83.0	62.0

*: Statistically significant (*p* < 0.05) between groups in T test. *Ns:* no statistical differences found. WT: Wilcoxon rank sum test.

**Table 4:** Redox biomarkers clustered by plasmatic levels of vitamin A and E.

	Vitamin A (mcg/dL)			
	Deficiency (<42) (*n* = 16)	Normal (42–68) (*n* = 55)	High (>68) (*n* = 26)	ANOVA Test
MDA (ng/mL)	421.9	410.67	424.88	
GSH/GSSG	0.38	0.36	0.37	0.95
TAC (mM)	2.680	3.449	3.178	0.13
SOD-3 (U/mL)	1.081	1.630	1.273	0.28
NOX (µg/mL)	26.8	33.0	34.5	0.11
	Vitamin E (mcg/mL)			
	Deficiency (<8.6) (*n* = 1)	Normal (8.6–13) (*n* = 21)	High (>13) (*n* = 75)	ANOVA test
MDA (ng/mL)	442.0	379.6	426.3	0.16
GSH/GSSG	0.16	0.37	0.36	0.46
TAC (mM)	1.611 *	2.949 *	3.355 *	***p* = ** **0.02**
SOD-3 (U/mL)	0	1.289	1.444	0.35
NOX (µg/mL)	26.9	31.3	32.8	0.83

* Statistically significant (*p* < 0.05) between groups in ANOVA test.

**Table 5:** Redox biomarkers clustered by colonization status and parasitic species.

	Colonized (*n* = 48)		Non-Colonized (*n* = 49)		*t* Test	*B/DF* * (*n* = 39) Mean	*Gi* (*n* = 9) Mean	*t* Test
	Mean	SD	Mean	SD				
MDA (ng/mL)	408.5	72.4	424.0	103.4	0.53	402.1	436.2	0.46
GSH/GSSG	0.33 *	0.2	0.40 *	0.2	*p* = 0.034 *	0.33	0.33	0.53
TAC (mM)	3.57 *	1.1	2.93 *	1.1	*p* = 0.009 *	3.69	3.11	0.16
SOD-3 (U/mL)	1.65	1.2	1.24	1.1	0.10	1.81	0.97	0.05
NOX (µg/mL)	29.9	15.2	34.7	18.0	0.34	29.9	30.2	0.98
VitA (µg/dL)	55.1	12.1	60.3	23.9	0.65	56.5	49.3	0.17
VitE (µg/mL)	15.8	3.8	17.1	5.3	0.34	15.9	15.4	0.75

*SD*: standard deviation *: Statistically significant (*p* < 0.05) between groups in T test. *B/DF*: *Blastocystis* sp. and/or *D. fragilis* positives after excluding subjects co-infected with *G. intestinalis*. Gi: subjects positive for *G. intestinalis.*

**Table 6:** Redox biomarkers clustered by insulin resistance and presence of NAFLD.

	Insulin-Sensitivity Status ^1^		*t* Test	Fatty Liver Disease Status ^2^		
	Resistant (*n* = 59)	Sensitive (*n* = 28)		NAFLD + (*n* = 69)	NAFLD - (*n* = 23)	*t* Test
MDA (ng/mL)	411.1	407.9	0.82	412.2	420.3	0.25
GSH/GSSG	0.388 *	0.297 *	*p* = 0.011 *	0.383 *	0.303 *	*p* = 0.045 *
TAC (mM)	3.29	3.16	0.59	3.28	3.15	0.60
SOD-3 (U/mL)	1.66 *	1.04 *	*p* = 0.025 *	1.47	1.35	0.61
NOX (µg/mL)	36.7 *	25.3 *	*p* = 0.01 *	34.9 *	24.5 *	*p* = 0.002 *

^1^ After excluding 10 participants with type 2 diabetes treated with insulin. ^2^ Data regarding NAFLD was missing from 5 subjects. * Statistically significant (*p* < 0.05) between groups.

**Table 7:** Redox biomarkers with respect to the presence of insulin resistance and colonization status.

	Insulin-Resistant			Insulin-Sensitive		
	Colonized (*n* = 30)	Non-Colonized (*n* = 29)	T Test	Colonized (*n* = 15)	Non-Colonized (*n* = 11)	T Test
MDA (ng/mL)	414.5	407.7	0.78	396.6	426.1	0.22
GSH/GSSG	0.33 *	0.45 *	*p* = 0.007 *	0.33	0.26	*p* = 0.024 *
TAC (mM)	3.59 *	2.97 *	*p* = 0.022 *	3.64 *	2.54 *	*p* = 0.04 *
SOD-3 (U/mL)	1.84	1.48	0.25	1.12	0.82	0.43
NOX (µg/mL)	33.3	39.8	0.19	25.4	24.8	0.89

* Statistically significant (*p* < 0.05) between groups.

**Table 8:** Correlation between redox biomarkers and biochemical/anthropometric measurements.

Correlation	Estimate	Confidence Interval	*p*-Value
MDA and HOMA-IR	10.08	[3.5, 16.67]	0.003
MDA and plasmatic leptin	−0.92	[−1.70, −0.15]	0.020
MDA and plasmatic Vitamin A	−1.7	[−2.92, −0.48]	0.007
NOX and plasmatic ferritin	0.043	[0.005, 0.081]	0.028
SOD-3 and plasmatic C-RP levels	0.05	[0.007, 0.096]	0.025
TAC and % fat mass	0.123	[−0.247, 0.001]	0.052

## Data Availability

The data are contained within this article and supplementary material.

## Supplementary Materials

The following supporting information can be downloaded at: https://www.mdpi.com/article/10.3390/antiox11112090/s1, Table S1: Anthropometric results by bioimpedance analysis in regards of colonization; Table S2: Daily intake of antioxidant nutrients clustered by colonization status; Table S3: Redox parameters clustered in regards of the fulfilment of DRI of antioxidants.
