# Supplementary material for: Do Intestinal Unicellular Parasites Have a Role in the Inflammatory and Redox Status among the Severely Obese?
Source: Antioxidants (Basel). 2022 Oct 23;11(11):2090. doi: 10.3390/antiox11112090 (PMC9686585; doi:10.3390/antiox11112090)
Supplement: Supplementary file 1 [file antioxidants-11-02090-s001.zip › antioxidants-1954521-supplementary.pdf]

Table S1. Anthropometric results by bioimpedance analysis in regards of colonization.

	Colonized		Non-colonized		T test
	Mean	*SD*	Mean	*SD*	
BMI (kg/m^2)	46.2	*6.3*	45.2	*6.7*	*Ns*
Abdominal circumference (cm)	132.7	*13.3*	128.7	*14.2*	*Ns*
FM%	50.1	*4.9*	50.3	*5.2*	*Ns*
FFM%	49.9	*4.9*	49.7	*5.2*	*Ns*
SKM%	27.8	*2.9*	27.7	*3.2*	*Ns*
Visceral fat area (cm^2)	260.4	*30.2*	257.9	*35.8*	*Ns*
Phase angle	5.3	*0.7*	5.4	*0.7*	*Ns*

SD = standard deviation; BMI= body mass index; FM%: fat mass %; FFM%; fat free mass %, SKM%, skeletal muscle mass %; VFA: visceral fat area. *Ns*: no statistical differences found between groups.

Table S2. Daily intake of antioxidant nutrients clustered by colonization status.

	Colonized (*n* = 48)		Non-colonized (*n* = 49)		*WT*
	Median	1Q, 3Q	Median	1Q, 3Q	
Vitamin A (µg)	702.0	*536.0, 903.0*	688.0	*484.8, 859.5*	*0.13*
Vitamin C (mg)	87.3	*58.3, 118.0*	85.7	*57.9, 118.0*	*0.21*
Vitamin E (mg)	5.5	*4.0, 8.3*	5.5	*3.6, 8.1*	*0.06*
ω3/ω6 ratio	0.17	*0.13, 0.28*	0.18	*0.13, .27*	*0.55*
Copper (mg)	1.2	*0.9, 2.1*	1.2	*0.9, 1.8*	*0.23*
Zinc (mg)	8.5	*6.6, 11.2*	8.0	*6.4, 10.5*	*0.06*
Manganese (mg)	2.7	*2.1, 6.5*	2.6	*2.0, 5.4*	*0.37*
Selenium (µg)	104.0	*77.6, 138.0*	105.5	*77.9, 135.5*	*0.34*

1Q, 3Q: first and third quartiles *Statistically significant ($p < 0.05$) between groups. WT: Wilcoxon rank sum test.

Table S3. Redox parameters clustered in regards of the fulfilment of DRI of antioxidants.

	None (*n* = 32)	Vitamin A (*n* = 4)	Vitamin C (*n* = 23)	Vitamin A + C (*n* = 22)	Vitamin A+C+E (*n* = 3)	KW T
MDA (ng/mL)	418.3	450.0	401.2	388.1	431.7	*Ns*
GSH/GSSG	0.38	0.48	0.35	0.37	0.33	*Ns*
TAC (mM)	3.31	3.59	3.26	3.24	3.84	*Ns*
SOD-3 (U/mL)	1.47	1.41	1.36	1.48	0.93	*Ns*
NOX (µG/mL)	30.3	33.9	37.4	33.0	27.0	*Ns*

KWT= Kruskal-Wallis test. *Ns:* no statistical differences found.
